# Comparison between pericapsular nerve group (PENG) block with lateral femoral cutaneous nerve block and supra-inguinal fascia iliaca compartment block (S-FICB) for total hip arthroplasty: a randomized controlled trial

**DOI:** 10.1007/s00540-023-03192-6

**Published:** 2023-04-12

**Authors:** Ludan Liang, Can Zhang, Wei Dai, Kaihua He

**Affiliations:** Department of Anesthesiology, The First Affiliate Hospital of Chongqing Medical University, Chongqing, China

**Keywords:** Total hip arthroplasty, Nerve block, Analgesia

## Abstract

**Purpose:**

To assess the efficacy of pericapsular nerve group (PENG) block combined with lateral femoral cutaneous nerve (LFCN) block in controlling postoperative pain and promoting recovery of lower extremity after total hip arthroplasty (THA), and to compare its effectiveness with supra-inguinal fascia iliaca compartment block (S-FICB).

**Materials and methods:**

92 patients undergoing THA with general anesthesia were randomly allocated to receive either a PENG with LFCN block (*n* = 46) using 30 ml 0.33% ropivacaine (20 ml for PENG block, 10 ml for LFCN block), or an S-FICB (*n* = 46) using 30 ml 0.33% ropivacaine. The primary outcome was the time to first postoperative walk. The secondary outcomes included intraoperative remifentanil consumption, postoperative hip flexion degree and muscle strength of the operative lower limbs in the supine position, pain scores (static and dynamic), rescue analgesia, postoperative nausea and vomiting (PONV), and nerve block-related complications.

**Results:**

The combination of PENG with LFCN blocks resulted in an earlier first postoperative walking time (19.6 ± 9.6 h vs 26.5 ± 8.2 h, *P* < 0.01), greater postoperative hip flexion degree at 6 h, 24 h and 48 h (all *P* < 0.01), and higher muscle strength of the operative lower limbs at 6 h after surgery (*P* = 0.03) compared to S-FICB. The difference in pain scores (static and dynamic) was only statistically significant at 48 h (*P* < 0.05). There were no differences in the other outcomes.

**Conclusions:**

PENG with LFCN blocks is more effective than S-FICB in shortening the time to first postoperative walk and preservation hip motion after THA, which makes it a suitable addition to enhanced recovery programs following surgery.

## Introduction

Total hip arthroplasty (THA) is a widely used method for treating hip fractures, femoral head necrosis, and other diseases. Early postoperative lower limb functional exercise and ambulation after THA are important for relieving pain and reducing the risk of complications such as deep vein thrombosis [1, 2]. The anterior part of hip capsule, which is the source of pain in the hip joint, is mainly innervated by the articular branch of the femoral nerve and the obturator nerve [3, 4]. Fascia iliaca compartment block (FICB) is commonly used for analgesia in THA, and supra-inguinal fascia iliaca compartment block (S-FICB) is recommended due to its superior analgesic efficacy compared to the classical approach [5, 6]. However, the effectiveness of obturator nerve block in S-FICB is unclear, leading to inadequate medial hip analgesia [7]. Additionally, FICB can cause early postoperative quadriceps weakness due to motor block of the femoral nerve [8, 9], which can affect recovery programs. Pericapsular nerve group block (PENG) has been shown to significantly relieve hip fracture pain and provide effective postoperative analgesia for THA [10, 11], while preserving quadriceps muscle strength [12]. However, the presence of skin incision and subcutaneous dissections of THA are typically located on the lateral thigh, which is supplied by the lateral femoral cutaneous nerve (LFCN). Therefore, some researchers have suggested combining PENG block with LFCN block to provide better analgesia effect than the PENG block alone [13].

At present, there are no randomized controlled trials studying the efficacy of PENG block combined with LFCN block. This study aims to assess the efficacy of PENG block performed with LFCN block in controlling postoperative pain and promoting motor function recovery and to compare its effectiveness with S-FICB. We hope that the results of this study will provide a clinical reference for the improvement of perioperative pain management and rehabilitation programs in THA.

## Materials and methods

### Recruitment and randomization

**Fig. 1:**
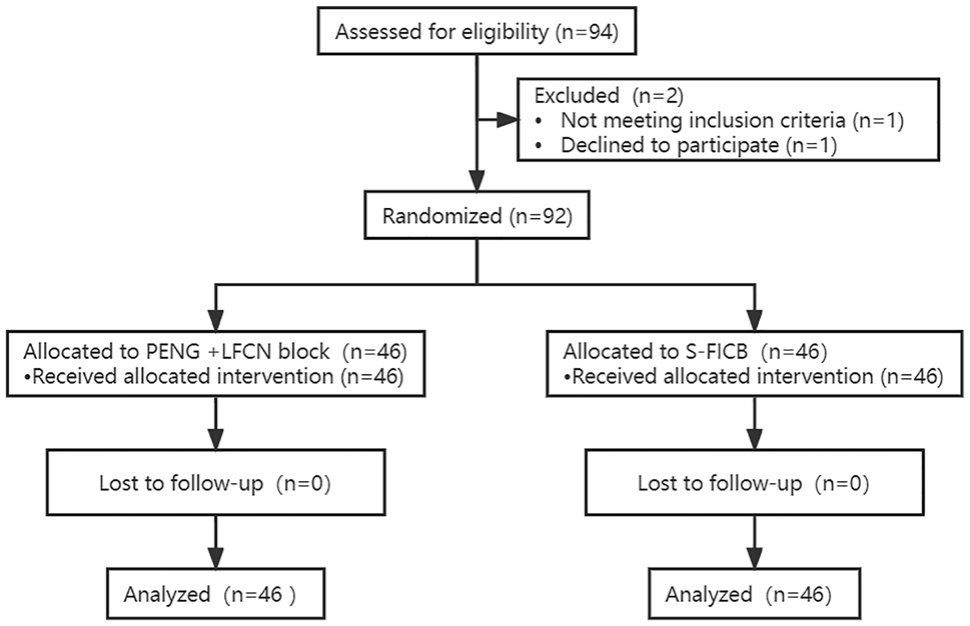
CONSORT diagram. PENG, pericapsular nerve group block; S-FICB, supra-inguinal fascia iliaca block

This study was registered in the Chinese Clinical Trial Registry (ChiCTR2100051521) and secured by the Ethics Committee of the First Affiliated Hospital of Chongqing Medical University on 6/16/2021. After signing the informed consent, a total of 92 patients undergoing elective one- sided THA were enrolled over a period of 9 months (11/10/2021 to 8/25/2022) (Fig. 1). Inclusion criteria were: age ≥ 18 years, American Society of Anesthesiologists (ASA) physical status I–III. Exclusion criteria included inability to consent to the study, weighed < 30 kg, allergy to local anesthetic (LA), coagulopathy, infection in the injection site, history of opioid dependence, obvious organ dysfunction (liver, kidney and other organs), decline of cognitive state, and inability to communicate.

All the subjects were randomly allocated to either the PENG + LFCN group or the S-FICB group in a 1:1 ratio using the random allocation scheme provided by RESEARCH-RANDOMIZE (https://www.randomizer.org/), based on the order in which patients were admitted. The random assignment was generated and kept by the assistant of our team and the patients and remaining researchers were not aware of the assignment. The group designation data were unblinded before the patients were treated with nerve block. The outcome data were collected by a blinded study investigator.

### Ultrasound-guided block procedures

Peripheral vein access was established after the patients entered operating room, and non-invasive blood pressure (NIBP), heart rate (HR), and peripheral capillary oxygen saturation (SpO_2_) were routinely monitored. All the patients received nasal cannula oxygen (2 L/min) and were placed in the supine position, the skin of operating area was routinely sterilized then. All the nerve blocks were performed by the same senior anesthesiologist before anesthesia induction and both groups were treated with 30 ml volume ropivacaine hydrochloride at 0.33% concentration for nerve block. During the performance of nerve blocks, the screen of ultrasound was ensured not to be in the patients’ field of vision.

**Fig. 2:**
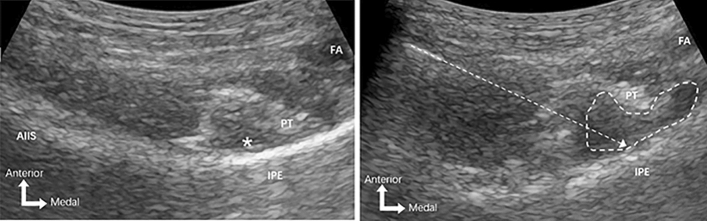
Images of ultrasound-guided pericapsular nerve group (PENG) block. The figure on the left shows the ultrasonic anatomy of PENG block; the needle tip was positioned between the psoas tendon and the pubic ramus using an in-plane approach. The figure on the right shows the local anesthetic spread following injection. Asterisk, target for local anesthetic injection; arrow, needle pathway; area circled by dashed line, local anesthetic spread; AIIS, anterior inferior iliac spine; IPE, iliopubic eminence; PT, psoas tendon; FA, femoral artery

**Fig. 3:**
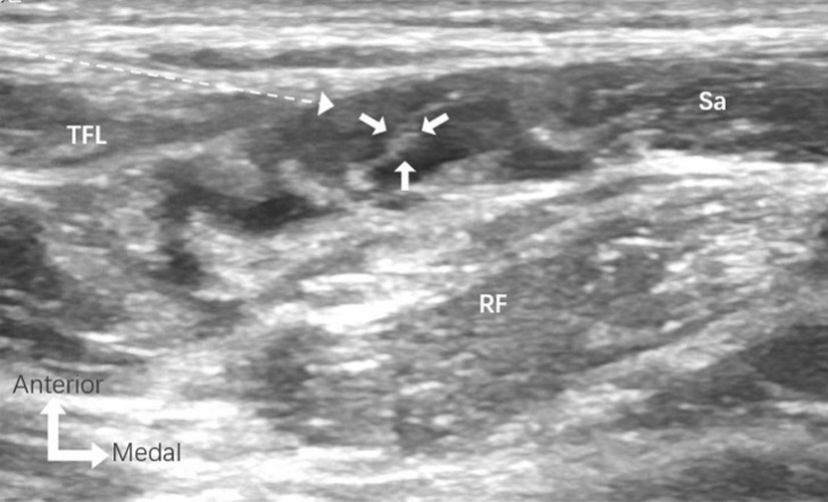
Images of ultrasound-guided lateral femoral cutaneous nerve (LFCN) block. The needle tip was positioned in the tunnel formed by the fascia between the sartorius and the tensor fascia lata. Dashed arrow, needle pathway; large arrows, LFCN; TFL, tensor fascia lata; RF rectus femoris; Sa, sartorius

PENG + LFCN block: The low-frequency curvilinear probe (3–5 MHz) of ultrasound (GE Logiq e, GE Healthcare, USA) was placed in a transverse plane over the anterior inferior iliac spine (AIIS) and aligned with the pubic ramus by rotating the probe counterclockwise to obtain a hyperechoic bright line, which is the iliopubic eminence (IPE). In this view, the iliopsoas muscle and psoas tendon, the femoral artery, and pectineus muscle were observed. Using the in-plane injection technique, a 22G, 80 mm insulated block needle was inserted in a lateral-to-medial direction, and the tip was placed between the psoas tendon anteriorly and the pubic ramus posteriorly. After no blood was drawn back, the LA (20 ml, 0.33% ropivacaine) was injected to get an image of the psoas tendon uplifted (Fig. 2). After the PENG block was performed, a high-frequency linear probe (6–12 MHz) was placed on the inguinal ligament to get a short-axial view of femoral artery, then the probe was moved laterally to identify the sartorius muscle, the tail of the probe was positioned toward the anterior superior iliac spine to observe the LFCN covered by fascia between sartorius and tensor fascia lata. After no blood was drawn back, the LA (10 ml, 0.33% ropivacaine) was injected following negative aspiration (Fig. 3).

**Fig. 4:**
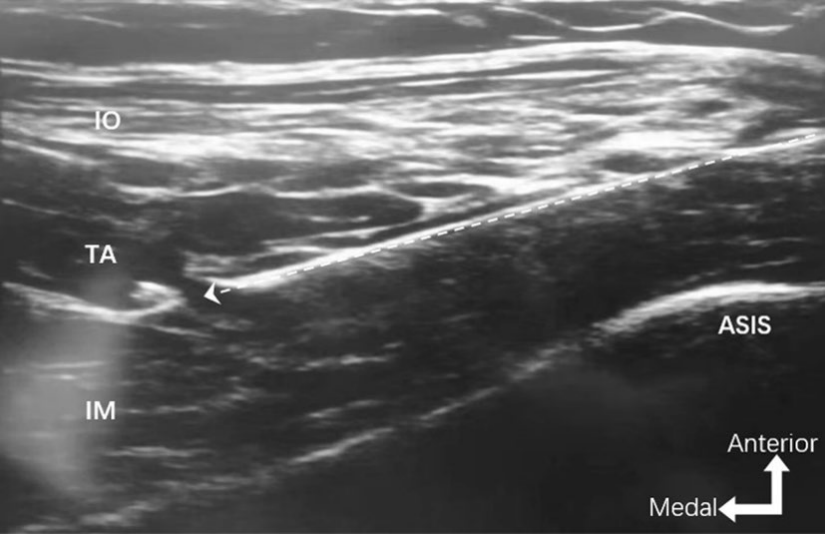
Images of ultrasound-guided supra-inguinal fascia iliaca block (S-FICB). The needle tip was located under the fascia iliacus between the transversal abdominus muscle and iliac muscle. Dashed arrow, needle pathway. IO internal oblique muscle, TA transverse abdominus muscle, IM iliacus muscle, ASIS anterior superior iliac spine

S-FICB: A high-frequency linear probe (6–12 MHz) of ultrasound (GE Logiq e, GE Healthcare, USA) was used. The probe was placed adjacent to the inguinal ligament with its long axis parallel to the ligament. After the femoral artery and the femoral nerve was observed, the probe was moved laterally to identify the sartorius muscle and placed it at the center of the screen. Then the probe was moved cephalically to the anterior superior iliac spine (ASIS) until the image of the sartorius muscle disappeared, and the medial side of ASIS was identified as the iliacus muscle. Next, by rotating the medial end of the ultrasound probe toward the umbilicus, the ASIS, iliac bone, and abdominal muscles were observed on the screen. Using the in-plane technique, a 22G, 80 mm insulated block needle was inserted in a lateral-to-medial direction. When the needle tip penetrated below the fascia iliacus, the LA (30 ml, 0.33% ropivacaine) was injected following negative aspiration to obtain an image showing the LA spread between the iliacus muscle and the fascia iliacus (Fig. 4).

Pain scores with a straight leg raise of the affected limb to 15 degrees were assessed by the same blinded investigator before and 30 min after block performance. Subsequently, this investigator assessed the block of LFCN by testing for loss of pain sensation on the lateral thigh. Successful block performance was defined as a reduction in pain score compared to before the intervention and loss of pain sensation on the lateral thigh. Case of unsuccessful block performance were not included in the statistical analysis. If the nerve block was unsuccessful, the investigator who previously assessed the effects of the block would ask the surgeon to perform local infiltration analgesia (LIA) at the end of the surgical procedure, which involved injecting 20 ml 0.33% ropivacaine around the joint capsule and 10 ml 0.33% ropivacaine infiltrated around the wound.

### Anesthesia and postoperative analgesia

Following the nerve block, patients received general anesthesia administered by an anesthesiologist who was blinded to the allocation result. All the patients were treated with 10 mg of intravenous dexamethasone before surgery. For anesthesia induction, propofol (2.0–2.5 mg/kg), sufentanil (0.4–0.5 μg/kg), and vecuronium (0.1 mg/kg) were administered. Surgical interventions were performed by four teams of orthopedic surgeons using posterolateral approaches.

During the operation, propofol (2–5 mg/kg/h), remifentanil (0.1–0.5 μg/kg/min), and sevoflurane (1–2% concentration) were used for anesthesia maintenance at the discretion of the anesthesiologist. The bispectral index (BIS) was maintained between 40 and 60, and the fluctuation ranges of invasive systolic blood pressure (ISBP) and heart rate (HR) were kept within 20% of the preoperative level.

All the patients received 2 mg intravenous tropisetron at the end of the surgery, and patient-controlled intravenous analgesia (PCIA) was initiated before patients were sent to post-anesthesia care unit (PACU). The PCIA combination of drugs consisted of 800 mg of tramadol, 100 mg of flurbiprofen axetil, and 54 ml of normal saline, and with a total volume of 80 ml. The PCIA system was set to deliver 5 ml for the first dose, a background infusion rate of 1 ml/h, a patient-controlled dose of 2 ml, and a lockout time of 15 min. In addition to PCIA, the patients were treated with intravenous parecoxib sodium (40 mg per 24 h) for background pain control in the general ward during 48 h. Tramadol was administered orally for rescue analgesia when the pain score (VAS) ≥ 5, and the specific dose was evaluated by the ward surgeon.

### The primary outcome

Our primary outcome was “time to first walk”, defined as the duration between the end of surgery and the first time the patient was able to walk with the assistance of a walking aid under the guidance of a rehabilitation physician.

### The secondary outcomes

The secondary outcomes postoperatively included intraoperative remifentanil consumption, the degree of hip flexion on the operative side at 6 h, 24 h, and 48 h, lower limb muscle strength of the operative side at 6 h, 24 h, and 48 h, static and dynamic pain scores at 6 h, 24 h, and 48 h, the number of rescue analgesia, postoperative nausea and vomiting (PONV) at 48 h, and the rate of nerve block-related complications. The incidences of vascular puncture, paresthesia, and LA toxicity were also recorded by a supervisor during the block. Since the postoperative 12 h in this study were generally late at night, we only collected the outcomes at 6 h, 24 h, and 48 h to avoid disturbing the patients’ rest.

The hip flexion degree of the operative side was measured using angle gauge in supine position (to avoid hip dislocation due to hip flexion beyond 90°).

The lower limb muscle strength of the operative side was assessed using a manual muscle test (MMT) in supine position. The strength of quadriceps muscle group, iliopsoas muscle, sartorius muscle could be evaluated, mainly to evaluate the quadriceps muscle strength: Grade 0, no contraction; Grade 1, flickering contraction (no active knee extension and leg lift, only slight quadriceps contraction); Grade 2, full range of motion (ROM) with eliminated gravity (hip flexion, knee extension with eliminated gravity); Grade 3, full ROM with against gravity (leg lift and knee extension with against gravity but no resistance); Grade 4, full ROM with against gravity with minimal resistance (leg lift and knee extension with against gravity and minimal resistance); Grade 5: full ROM with against gravity with maximal resistance (leg lift and knee extension with against gravity and maximal resistance).

Static and dynamic pain scores was recorded at 6 h, 24 h, and 48 h (0, no pain; 10, worst imaginable pain).

### Sample size calculation

We used PASS 16.0 software to calculate the sample size. Based on our preliminary study, we estimated that the first postoperative walking time of the S-FICB group would be 27.6 ± 12.3 h and the PENG + LFCN group would be 18.5 ± 12.3 h value. Under these assumptions, with *α* = 0.05 (two-sided) and *β* = 0.10, we calculated that the minimum sample size of our study should be approximately 80. To account for a 10–15% protocol deviation and violation, we planned to recruit 94 patients.

### Statistical analysis

We performed statistical analysis using IBM SPSS Statistics 20.0 software. Continuous variables with normal distribution were expressed as mean (SD) or percentage (%), and we used median (range) to express continuous variables that presented skewed distribution. Student’s *t* test or Mann–Whitney *U* test was used for comparison according to the feasibility of the normal assumption of continuous variables. Categorical variables expressed as incidences were compared using *χ*^2^ test or Fisher’s exact tests probability. *P* < 0.05 was considered statistically significant between groups.

## Results

After obtaining written informed consents, a total of 92 patients undergoing THA were included in the final analysis (Fig. 1). The demographic characteristics of the two groups were comparable. Their surgery durations and lengths of PACU stay were also comparable (Table 1).

**Table 1:** Patient characteristics

	PENG + LFCN	S-FICB
	(*n* = 46)	(*n* = 46)
Sex, *n* (%)		
Male	18 (39)	19 (41)
Female	28 (61)	26 (59)
Age (years)	66.7 (14.4)	67.3 (10.2)
BMI (kg/m^2^)	23.3 (3.7)	24.3 (3.6)
ASA class, *n* (%)		
II	26 (57)	24 (52)
III	20 (43)	21 (48)
Preoperative diagnosis, *n* (%)		
Fracture	13 (28)	12 (26)
No fracture	33 (72)	33 (74)
Surgery duration (min)	48.2 (12.1)	50.6 (14.5)
Duration of PACU stay (min)	45.3 (11.2)	43.6 (10.4)

Values are mean (SD) or number (proportion)

*ASA* American Society of Anesthesiologists, *BMI* body mass index, *PENG + LFCN* pericapsular nerve block with lateral femoral cutaneous nerve block, *S-FICB* supra-inguinal fascia iliaca block

Compared to the S-FICB group, the PENG + LFCN group demonstrated a significantly earlier time to first postoperative walk (19.6 ± 9.6 h vs 26.5 ± 8.2 h, *P* < 0.01), as well as significantly higher hip flexion degrees at all time points (all *P* < 0.01) (Table 2). However, the only statistically significant difference in lower limb muscle strength of the operative side between the two groups was at 6 h postoperatively (2[1–5] vs 2[0–4], *P* = 0.03). In terms of frequency distribution, the PENG + LFCN group exhibited a positively skewed distribution, with more than 70% of patients having muscle strength ≥ grade 2, while the S-FICB group demonstrated a negatively skewed distribution, with more than 70% of patients having muscle strength ≤ grade 2. Therefore, the postoperative lower limb muscle strength was better in the PENG + LFCN group than in the S-FICB group (Table 2).

**Table 2:** Postoperative lower limb motor function-related outcomes

	PENG + LFCN	S-FICB	Median difference (95% CI)	*P* values
	(*n* = 46)	(*n* = 46)		
Time to first walk (h)	19.6 (9.6)	26.5 (8.2)	− 6 (− 8 to − 3)	< 0.01
Hip flexion degrees (°)				
*T*_1_	27.9 (15.4)	16.1 (17.5)	15 (10–20)	< 0.01
*T*_2_	42.2 (14.7)	30.7 (18.0)	13 (7–19)	< 0.01
*T*_3_	51.1 (14.0)	41.9 (15.9)	10 (5–15)	< 0.01
Muscle strength (grade)				
*T*_1_	2 (1–5)	2 (0–4)	0 (0–1)	0.03
*T*_2_	3 (1–5)	3 (1–4)	0 (0–0)	0.65
*T*_3_	3 (2–5)	4 (2–5)	0 (− 1 to 0)	0.13

Continuous variables are presented as mean (SD); ordinal variables (muscle strength) are presented as median (range), the median difference is presented as median (PENG + LFCN) – median (S-FICB) and 95% CI

*T*_*1*_ postoperative 6 h, *T*_*2*_ postoperative 24 h, *T*_*3*_ postoperative 48 h, *PENG + LFCN* pericapsular nerve block with lateral femoral cutaneous nerve block, *S-FICB* supra-inguinal fascia iliaca block

**Fig. 5:**
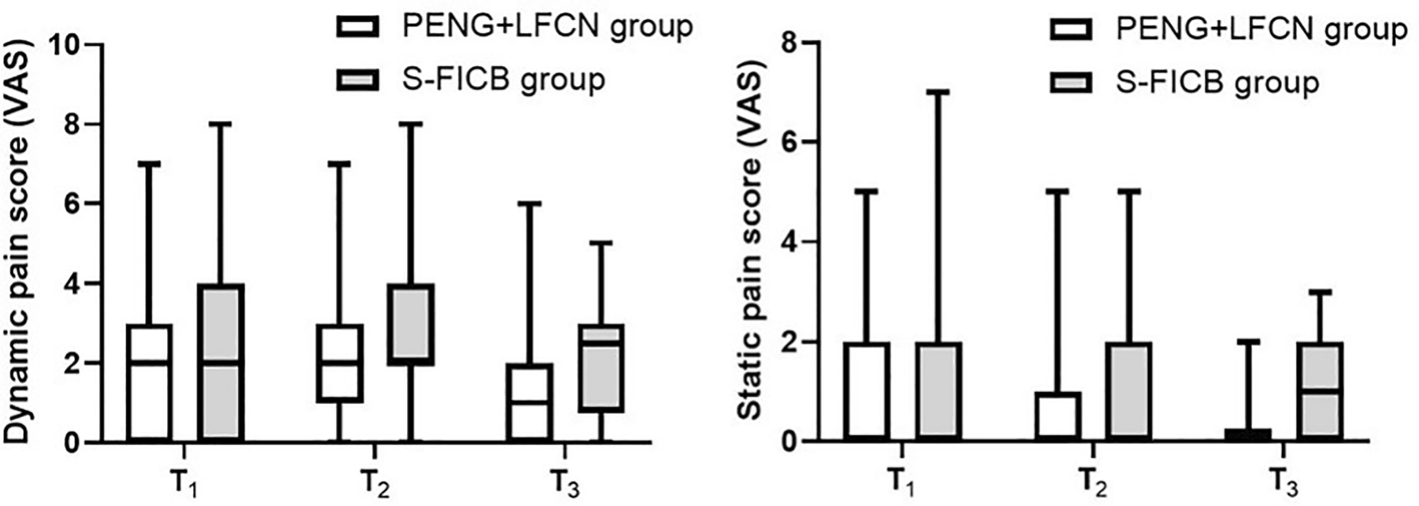
Pain score (VAS) outcomes, presented as median [(IQR) range]. Mann–Whitney *U* test of variance only detected statistically significant effects between the groups at *T*_3_ (static pain scores, *P* < 0.01; dynamic pain scores, *P* = 0.04). *T*_1_ postoperative 6 h, *T*_2_ postoperative 24 h, *T*_3_ postoperative 48 h, PENG + LFCN pericapsular nerve block with lateral femoral cutaneous nerve block, S-FICB supra-inguinal fascia iliaca block

However, the difference in postoperative pain scores (dynamic and static) between the two groups was only statistically significant at 48 h (all *P* < 0.05) (Fig. 5). There was no significant difference in intraoperative remifentanil consumption between the two groups, and the rate of rescue analgesia at 48 h postoperatively was comparable between the groups (Table 3).

**Table 3:** Intraoperative opioid consumption and other postoperative outcomes

	PENG + LFCN	S-FICB	*P* values
	(*n* = 46)	(*n* = 46)	
Intraoperative remifentanil consumption (µg)	357.57 (62.4)	370.41(58.5)	0.31
Rescue analgesic, *n* (%)	6 (13)	9 (20)	0.39
PONV, *n* (%)	2 (4)	2 (4)	> 0.99
Postoperative hip infection, *n* (%)	0 (0)	0 (0)	> 0.99
Vascular puncture, *n* (%)	0 (0)	0 (0)	> 0.99
Paresthesia, *n* (%)	0 (0)	0 (0)	> 0.99
LAST, *n* (%)	0 (0)	0 (0)	> 0.99

Values are presented as number (proportion)

*PONV* postoperative nausea and vomiting, *LAST* local anesthetic systemic toxicity, *PENG + LFCN* pericapsular nerve block with lateral femoral cutaneous nerve block, *S-FICB* supra-inguinal fascia iliaca block

Postoperative nausea and vomiting did not differ between the two groups. No postoperative complications related to nerve block were recorded in either group (Table 3).

## Discussion

According to previous studies, preoperative FICB or PENG block can significantly reduce postoperative pain scores in elderly patients with hip fracture, compared with no block [14–17]. In our study, we also observed that preoperative nerve block can significantly benefit postoperative analgesia in these patients. Therefore, it is necessary to include these patients in the intervention.

Early mobility is an important component of the Enhanced Recovery After Surgery (ERAS) theory. Scholars recommend that on-and-off bed functional exercise can be performed on the day of surgery, and patients can be guided to train lower limb function and get out of bed as early as 4 h postoperatively [18, 19]. Ambulation with physical therapy began within 6 h of surgery in the fast-track pathway. Fast-track THA can shorten the length of hospital stay by about 1–2 days compared with the traditional pathway [20]. Another study also showed that early mobility of THA on day of operation reduced length of stay by 0.2 days, and most patients can be discharged within 3 days or less after surgery compared to initiating physiotherapy on day 1 after surgery [1]. In our preliminary study, we observed that the decision of the surgeons and the intention of the patient greatly interfered with the discharge time, leading to the non-objectivity of the results. Therefore, we did not compare the length of postoperative hospital stay between the two groups. In our results, although the time to first walk in the PENG + LFCN group was 8 h earlier than that in the S-FICB group, the mean times were both on the day 1 after surgery. A tentative inference from this result is that this time gap of 8 h may not significantly affect the entire rehabilitation process after THA or even shorten the length of hospital stay. This inference has also been verified to some extent in G. Pascarella's study. The mean time of first postoperative walk of patients undergoing PENG block in THA was 22.1 ± 9.6 h, which was about 10 h earlier than that in the non-block group. However, there was no significant difference in length of stay between the two groups [17].

Although PENG block has the advantages of sparing quadriceps strength and faster knee function recovery than non-block group [14], a study comparing S-FICB and PENG block for THA suggested that the advantage of PENG block on quadriceps strength sparing was only reflected within 6 h after surgery, and PENG block had no benefit in terms of length of hospital stay [21]. Similarly, in our study, PENG combined with LFCN block was superior to S-FICB in lower limb muscle strength only at 6 h postoperatively. It is possible that with the extension of time, the motion block effect of low concentration ropivacaine (0.33% concentration) basically disappeared after 6 h postoperatively, the meta-analysis showed that FICB did not increase the risk of falls [22]. Therefore, we speculate that the motion block of S-FICB only exists for a short period after surgery.

It is reasonable to assume that a short period of quadriceps strength sparing after surgery may not be sufficient to make a difference in the overall postoperative rapid recovery process. Therefore, compared with S-FICB, PENG combined with LFCN block may have no obvious clinical advantage in shortening bed time, but it can enhance the intensity of hip rehabilitation training and improve the range of hip motion, which is conducive to the patient's postural change and increase patient comfort.

A previous controlled trial showed that preoperative PENG block did not reduce postoperative pain scores compared with S-FICB [21]. Although pain scores and cumulative opioid consumption favored PENG group when compared to FICB group, this superiority only existed within 24 h postoperatively [23]. Conversely, the difference in pain scores of our study was only found at 48 h, and PENG block alone can significantly reduce pain scores within 48 h postoperatively compared with placebo [17]. Based on this, we speculate that the analgesic effect of PENG with LFCN block is better than that of PENG block alone, and the duration of the analgesic effect may be longer than S-FICB. However, this difference may be due to our insufficient sample size. Most of the patients included in this study were the elderly and had slower metabolism of local anesthetics. Therefore, it may be necessary to include more samples or specifically analyze the influence of the two blocks on elderly patients with THA.

Our study has some limitations. First, according to the anatomical differences in the distribution of superficial sensory nerve, the posterior incision extends beyond the territory of the LFCN to the subcostal territory and may also involve the lateral cutaneous branch of the iliohypogastric nerve [24]. For this reason, the use of LFCN block for surgical incision analgesia alone may not be sufficient for some patients in this study. The use of PCIA and other analgesics may have masked this insufficiency. Second, some clinical cadaveric studies favored the use of high amounts of local anesthetic (40 ml) for FICB [15, 21, 25, 26]. Considering the safety of nerve block, we chose 30 ml 0.33% ropivacaine for FICB based on 95% effective volume (EV95) of 0.25% ropivacaine [27], and chose 20 ml local anesthetics for PENG block according to Giron-Arango’s recommendation [10]. However, it has been found that PENG block with 20 ml of LA did not seem to completely circumvent motor block, which may have affected our postoperative outcome to some extent. In addition, to accurately quantify the quadriceps muscle strength, a dynamometer is needed [14]. However, our department could not provide a dynamometer. Instead, we measured the lower extremity muscle strength of the operative side to reflect the postoperative motor function. Another limitation of this study is the potential impact of surgeon decision-making on postoperative outcomes. Surgeons may require patients to perform rehabilitation exercises only in bed for the first 24 h after surgery due to concerns that early walking could lead to hip dislocation. Furthermore, the first postoperative walk must be supervised by the guidance of rehabilitation physicians. This may result in differences between the time when rehabilitation doctors arrive at the ward to guide patients to walk and the time when patients can actually walk. Therefore, our current approach to rehabilitation training after THA may not fully realize the benefits of PENG with LFCN block on motor function protection for patients. Although PENG with LFCN blocks can shorten the time to first postoperative walk compared to S-FICB, the time difference is small and may not have significant clinical advantages.

In conclusion, PENG with LFCN blocks can provide motion sparing advantages, leading to better preservation of hip motion postoperatively. This can facilitate more intense joint rehabilitation training in the early postoperative period. Although the motion sparing advantage of PENG blocks may only be maintained for a short period after surgery, it is still beneficial for patients.

## Data Availability

The data that support the findings of this study are available from the corresponding author upon reasonable request.
